# (*E*)-2-(2,3-Dimethyl­anilino)-*N*′-(thio­phen-2-yl­methyl­idene)benzohydrazide

**DOI:** 10.1107/S160053681203259X

**Published:** 2012-07-25

**Authors:** Hoong-Kun Fun, Tze Shyang Chia, Mashooq A. Bhat, Mohamed A. Al-Omar, Hatem A. Abdel-Aziz

**Affiliations:** a X-ray Crystallography Unit, School of Physics, Universiti Sains Malaysia, 11800 USM, Penang, Malaysia; b Department of Pharmaceutical Chemistry, College of Pharmacy, King Saud University, PO Box 2457, Riyadh 11451, Saudi Arabia

## Abstract

In the title compound, C_20_H_19_N_3_OS, the central benzene ring makes dihedral angles of 45.36 (9) and 55.33 (9)° with the thio­phene ring and the dimethyl-substituted benzene ring, respectively. The dihedral angle between the thio­phene ring and dimethyl-substituted benzene ring is 83.60 (9)°. The thio­phene ring and the benzene ring are twisted from the mean plane of the C(=O)—N—N=C bridge [maximum deviation = 0.0860 (13) Å], with dihedral angles of 23.86 (9) and 24.77 (8)°, respectively. An intra­molecular N—H⋯O hydrogen bond generates an *S*(6) ring. In the crystal, mol­ecules are linked by N—H⋯O and C—H⋯O hydrogen bonds to the same acceptor atom, forming sheets lying parallel to the *bc* plane. The crystal packing also features C—H⋯π inter­actions.

## Related literature

For background to the chemistry and biological activity of diaryl amines, see: Reddy *et al.* (2010 ▶). For related structures, see: Bhat *et al.* (2012*a* ▶,*b* ▶,*c* ▶); Wang *et al.* (2010 ▶); Tian *et al.* (2010 ▶). For hydrogen-bond motifs, see: Bernstein *et al.* (1995 ▶). For reference bond-length data, see: Allen *et al.* (1987 ▶). For the stability of the temperature controller used for the data collection, see: Cosier & Glazer (1986 ▶).
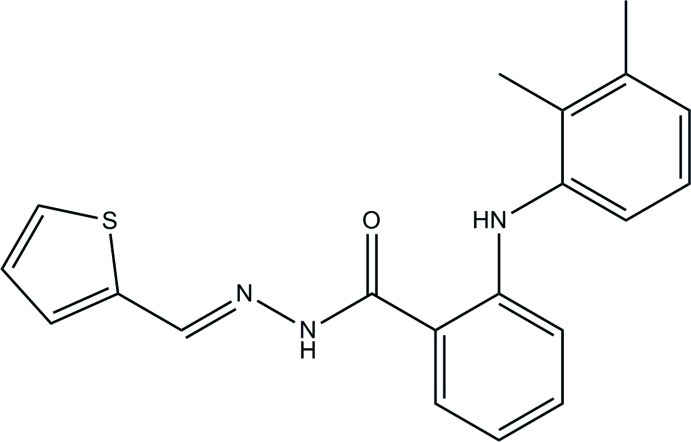


## Experimental

### 

#### Crystal data

C_20_H_19_N_3_OS
*M* *_r_* = 349.44
Monoclinic, 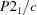

*a* = 14.0922 (14) Å
*b* = 15.9682 (15) Å
*c* = 8.1338 (8) Å
β = 105.344 (2)°
*V* = 1765.1 (3) Å^3^
*Z* = 4
Mo *K*α radiation
μ = 0.20 mm^−1^
*T* = 100 K
0.34 × 0.07 × 0.04 mm

#### Data collection

Bruker APEX DUO CCD diffractometer
Absorption correction: multi-scan (*SADABS*; Bruker, 2009 ▶) *T*_min_ = 0.936, *T*_max_ = 0.992
14626 measured reflections
5082 independent reflections
3338 reflections with *I* > 2σ(*I*)
*R*_int_ = 0.067

#### Refinement

*R*[*F* ^2^ > 2σ(*F* ^2^)] = 0.051
*wR*(*F* ^2^) = 0.128
*S* = 1.01
5082 reflections
236 parameters
H atoms treated by a mixture of independent and constrained refinement
Δρ_max_ = 0.37 e Å^−3^
Δρ_min_ = −0.35 e Å^−3^

### 

Data collection: *APEX2* (Bruker, 2009 ▶); cell refinement: *SAINT* (Bruker, 2009 ▶); data reduction: *SAINT*; program(s) used to solve structure: *SHELXTL* (Sheldrick, 2008 ▶); program(s) used to refine structure: *SHELXTL*; molecular graphics: *SHELXTL*; software used to prepare material for publication: *SHELXTL* and *PLATON* (Spek, 2009 ▶).

## Supplementary Material

Crystal structure: contains datablock(s) global, I. DOI: 10.1107/S160053681203259X/hb6898sup1.cif

Structure factors: contains datablock(s) I. DOI: 10.1107/S160053681203259X/hb6898Isup2.hkl

Supplementary material file. DOI: 10.1107/S160053681203259X/hb6898Isup3.cml

Additional supplementary materials:  crystallographic information; 3D view; checkCIF report

## Figures and Tables

**Table 1:** Hydrogen-bond geometry (Å, °) *Cg*1 and *Cg*2 are the centroids of the S1/C15–C18 and C1–C6 rings, respectively.

*D*—H⋯*A*	*D*—H	H⋯*A*	*D*⋯*A*	*D*—H⋯*A*
N2—H1*N*2⋯O1^i^	0.89 (2)	1.96 (2)	2.808 (2)	160 (2)
N1—H1*N*1⋯O1	0.85 (2)	2.02 (3)	2.704 (2)	137 (2)
C1—H1*A*⋯O1^ii^	0.95	2.58	3.410 (2)	146
C3—H3*A*⋯*Cg*1^iii^	0.95	2.98	3.732 (2)	137
C9—H9*A*⋯*Cg*2^iv^	0.95	2.84	3.649 (2)	144

Symmetry codes: (i) 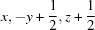; (ii) 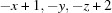; (iii) 
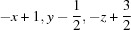
; (iv) 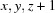.

**Fig. 1.**
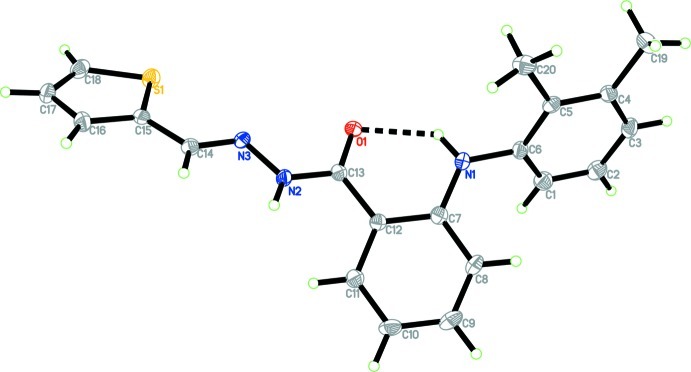
The molecular structure of the title compound with 50% probability displacement ellipsoids. The dashed line represents the intramolecular N—H···O hydrogen bond.

**Fig. 2.**
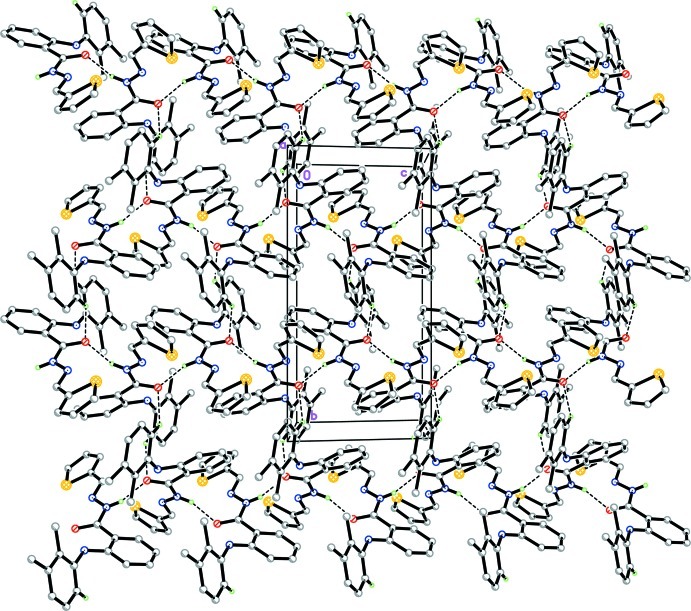
The crystal packing of the title compound. The dashed lines represent the hydrogen bonds. For clarity sake, hydrogen atoms not involved in hydrogen bonding have been omitted.

## supplementary crystallographic information

### Comment

In view of the importance of the chemistry and biological activity of diaryl amines (Reddy *et al.*, 2010) and in continuation to our interest in the chemistry of hydrazones (Bhat *et al.*, 2012*a*,*b*,*c*), we report herein the crystal structure of the title compound.

The asymmetric unit of the title compound is shown in Fig. 1. The central benzene ring [C7–C12] makes dihedral angles of 45.36 (9)° and 55.33 (9)° with the thiophene ring [S1/C15–C18] and dimethyl-substituted benzene ring [C1–C6], respectively. The dihedral angle between the thiophene ring and C1–C6 benzene ring is 83.60 (9)°. The thiophene ring and C7–C12 benzene ring are twisted from the mean plane of C13(═O1)—N2—N3═C14 bridge [maximum deviation = 0.0860 (13) Å at atom N3] with dihedral angles of 23.86 (9)° and 24.77 (8)°, respectively. An intramolecular N1—H1N1···O1 hydrogen bond generates an S(6) ring motif (Bernstein *et al.*, 1995) in the molecule. Bond lengths (Allen *et al.*, 1987) and angles are within normal ranges and are comparable to those found in related structures (Tian *et al.*, 2010; Wang *et al.*, 2010).

In the crystal (Fig. 2), molecules are linked by N2—H1N2···O1 and C1—H1A···O1 hydrogen bonds, with the same O atom acting as the acceptor, into sheets parallel to *bc* plane. The crystal packing also features C—H···π interactions (Table 1), involving *Cg*1 and *Cg*2 which are the centroids of S1/C15–C18 and C1–C6 rings, respectively.

### Experimental

The title compound was prepared by the reaction of thiophene-2-carbaldehyde (0.11 g, 1 mmol) and 2-[(2,3-dimethylphenylamine)]benzohydrazide (0.25 g, 1 mmol) in ethanol (25 ml). After stirring at room temperature for 3 h, the resulting mixture was concentrated under reduced pressure. The precipitate was washed with cold ethanol to afford the title compound. Yellow needles were recrystallized from ethanol solution by the slow evaporation of the solvent at room temperature after several days.

### Refinement

The N-bound H atoms were located in a difference Fourier map and refined freely [N—H = 0.85 (3) and 0.89 (2) Å]. The remaining H atoms were positioned geometrically [C—H = 0.95 and 0.98 Å] and refined using a riding model with *U*_iso_(H) = 1.2 or 1.5*U*_eq_(C). A rotating group model was applied to the methyl groups. Two outliers, (302) and (312), were omitted in the final refinement.

### Crystal data

**Table:**

C_20_H_19_N_3_OS	*F*(000) = 736
*M**_r_* = 349.44	*D*_x_ = 1.315 Mg m^−^^3^
Monoclinic, *P*2_1_/*c*	Mo *K*α radiation, λ = 0.71073 Å
Hall symbol: -P 2ybc	Cell parameters from 1664 reflections
*a* = 14.0922 (14) Å	θ = 2.9–24.3°
*b* = 15.9682 (15) Å	µ = 0.20 mm^−^^1^
*c* = 8.1338 (8) Å	*T* = 100 K
β = 105.344 (2)°	Needle, yellow
*V* = 1765.1 (3) Å^3^	0.34 × 0.07 × 0.04 mm
*Z* = 4	

### Data collection

**Table:**

Bruker APEX DUO CCD diffractometer	5082 independent reflections
Radiation source: fine-focus sealed tube	3338 reflections with *I* > 2σ(*I*)
Graphite monochromator	*R*_int_ = 0.067
φ and ω scans	θ_max_ = 30.0°, θ_min_ = 1.5°
Absorption correction: multi-scan (*SADABS*; Bruker, 2009)	*h* = −18→19
*T*_min_ = 0.936, *T*_max_ = 0.992	*k* = −18→22
14626 measured reflections	*l* = −11→11

### Refinement

**Table:**

Refinement on *F*^2^	Primary atom site location: structure-invariant direct methods
Least-squares matrix: full	Secondary atom site location: difference Fourier map
*R*[*F*^2^ > 2σ(*F*^2^)] = 0.051	Hydrogen site location: inferred from neighbouring sites
*wR*(*F*^2^) = 0.128	H atoms treated by a mixture of independent and constrained refinement
*S* = 1.01	*w* = 1/[σ^2^(*F*_o_^2^) + (0.0527*P*)^2^] where *P* = (*F*_o_^2^ + 2*F*_c_^2^)/3
5082 reflections	(Δ/σ)_max_ < 0.001
236 parameters	Δρ_max_ = 0.37 e Å^−^^3^
0 restraints	Δρ_min_ = −0.35 e Å^−^^3^

### Special details

**Table:**

Experimental. The crystal was placed in the cold stream of an Oxford Cryosystems Cobra open-flow nitrogen cryostat (Cosier & Glazer, 1986) operating at 100.0 (1) K.
Geometry. All e.s.d.'s (except the e.s.d. in the dihedral angle between two l.s. planes) are estimated using the full covariance matrix. The cell e.s.d.'s are taken into account individually in the estimation of e.s.d.'s in distances, angles and torsion angles; correlations between e.s.d.'s in cell parameters are only used when they are defined by crystal symmetry. An approximate (isotropic) treatment of cell e.s.d.'s is used for estimating e.s.d.'s involving l.s. planes.
Refinement. Refinement of *F*^2^ against ALL reflections. The weighted *R*-factor *wR* and goodness of fit *S* are based on *F*^2^, conventional *R*-factors *R* are based on *F*, with *F* set to zero for negative *F*^2^. The threshold expression of *F*^2^ > σ(*F*^2^) is used only for calculating *R*-factors(gt) *etc*. and is not relevant to the choice of reflections for refinement. *R*-factors based on *F*^2^ are statistically about twice as large as those based on *F*, and *R*-factors based on ALL data will be even larger.

### Fractional atomic coordinates and isotropic or equivalent isotropic displacement parameters (Å^2^)

**Table:**

	*x*	*y*	*z*	*U*_iso_*/*U*_eq_	
S1	0.10235 (4)	0.29345 (3)	0.79119 (6)	0.02004 (13)	
O1	0.44269 (10)	0.16844 (8)	0.95045 (15)	0.0164 (3)	
N1	0.63024 (13)	0.11358 (11)	1.0630 (2)	0.0221 (4)	
N2	0.38625 (12)	0.23505 (10)	1.1518 (2)	0.0152 (3)	
N3	0.30320 (11)	0.26223 (9)	1.03158 (19)	0.0152 (3)	
C1	0.72104 (15)	−0.01298 (12)	1.0366 (2)	0.0219 (4)	
H1A	0.6781	−0.0443	1.0854	0.026*	
C2	0.79626 (16)	−0.05289 (12)	0.9882 (3)	0.0225 (4)	
H2A	0.8060	−0.1114	1.0056	0.027*	
C3	0.85748 (15)	−0.00697 (12)	0.9138 (2)	0.0209 (4)	
H3A	0.9097	−0.0344	0.8817	0.025*	
C4	0.84361 (14)	0.07824 (12)	0.8855 (2)	0.0173 (4)	
C5	0.76785 (14)	0.12003 (11)	0.9365 (2)	0.0164 (4)	
C6	0.70796 (14)	0.07299 (12)	1.0141 (2)	0.0171 (4)	
C7	0.60869 (14)	0.10490 (11)	1.2183 (2)	0.0170 (4)	
C8	0.67460 (15)	0.06586 (12)	1.3575 (2)	0.0203 (4)	
H8A	0.7349	0.0443	1.3442	0.024*	
C9	0.65339 (16)	0.05829 (12)	1.5130 (3)	0.0239 (4)	
H9A	0.6989	0.0310	1.6044	0.029*	
C10	0.56674 (16)	0.08987 (12)	1.5376 (2)	0.0233 (4)	
H10A	0.5523	0.0841	1.6447	0.028*	
C11	0.50153 (15)	0.13000 (11)	1.4038 (2)	0.0188 (4)	
H11A	0.4420	0.1519	1.4203	0.023*	
C12	0.52102 (14)	0.13927 (11)	1.2440 (2)	0.0150 (4)	
C13	0.44837 (13)	0.18131 (11)	1.1041 (2)	0.0142 (4)	
C14	0.25109 (14)	0.31569 (11)	1.0879 (2)	0.0163 (4)	
H14A	0.2759	0.3391	1.1983	0.020*	
C15	0.15535 (14)	0.34032 (11)	0.9847 (2)	0.0156 (4)	
C16	0.09047 (15)	0.39305 (12)	1.0313 (3)	0.0207 (4)	
H16A	0.1061	0.4246	1.1340	0.025*	
C17	−0.00236 (15)	0.39559 (13)	0.9101 (3)	0.0234 (4)	
H17A	−0.0560	0.4289	0.9222	0.028*	
C18	−0.00604 (15)	0.34505 (12)	0.7747 (3)	0.0227 (4)	
H18A	−0.0625	0.3390	0.6811	0.027*	
C19	0.90953 (17)	0.12491 (14)	0.7978 (3)	0.0294 (5)	
H19A	0.9586	0.0863	0.7748	0.044*	
H19B	0.9430	0.1704	0.8716	0.044*	
H19C	0.8698	0.1483	0.6901	0.044*	
C20	0.75020 (17)	0.21221 (12)	0.9033 (3)	0.0241 (4)	
H20A	0.7177	0.2355	0.9857	0.036*	
H20B	0.7081	0.2205	0.7875	0.036*	
H20C	0.8133	0.2407	0.9149	0.036*	
H1N2	0.3987 (16)	0.2551 (13)	1.257 (3)	0.024 (6)*	
H1N1	0.5850 (19)	0.1361 (15)	0.985 (3)	0.035 (7)*	

### Atomic displacement parameters (Å^2^)

**Table:**

	*U*^11^	*U*^22^	*U*^33^	*U*^12^	*U*^13^	*U*^23^
S1	0.0187 (3)	0.0212 (2)	0.0189 (2)	0.0013 (2)	0.00243 (18)	−0.00158 (19)
O1	0.0181 (7)	0.0193 (6)	0.0122 (6)	0.0016 (5)	0.0045 (5)	−0.0001 (5)
N1	0.0172 (9)	0.0300 (9)	0.0203 (9)	0.0093 (7)	0.0073 (7)	0.0073 (7)
N2	0.0136 (8)	0.0194 (8)	0.0116 (7)	0.0030 (6)	0.0013 (6)	−0.0016 (6)
N3	0.0127 (8)	0.0180 (7)	0.0134 (7)	0.0012 (6)	0.0008 (6)	0.0013 (6)
C1	0.0206 (10)	0.0214 (10)	0.0231 (10)	−0.0019 (8)	0.0047 (8)	0.0026 (8)
C2	0.0260 (11)	0.0164 (9)	0.0239 (10)	0.0038 (8)	0.0046 (9)	0.0002 (8)
C3	0.0191 (10)	0.0230 (10)	0.0201 (10)	0.0064 (8)	0.0044 (8)	−0.0023 (8)
C4	0.0163 (10)	0.0212 (9)	0.0141 (9)	−0.0002 (7)	0.0036 (7)	−0.0004 (7)
C5	0.0172 (10)	0.0169 (9)	0.0128 (9)	0.0003 (7)	−0.0001 (7)	−0.0004 (7)
C6	0.0136 (9)	0.0208 (9)	0.0163 (9)	0.0013 (7)	0.0029 (7)	−0.0004 (7)
C7	0.0164 (9)	0.0165 (9)	0.0174 (9)	0.0001 (7)	0.0036 (7)	−0.0006 (7)
C8	0.0159 (10)	0.0215 (9)	0.0203 (10)	0.0031 (8)	−0.0012 (8)	0.0011 (8)
C9	0.0267 (11)	0.0222 (10)	0.0171 (10)	0.0029 (9)	−0.0042 (8)	0.0003 (8)
C10	0.0319 (12)	0.0239 (10)	0.0126 (9)	0.0030 (9)	0.0030 (8)	0.0005 (8)
C11	0.0201 (10)	0.0188 (9)	0.0174 (9)	0.0025 (8)	0.0050 (8)	−0.0007 (7)
C12	0.0142 (9)	0.0157 (8)	0.0143 (9)	−0.0016 (7)	0.0025 (7)	−0.0005 (7)
C13	0.0127 (9)	0.0142 (8)	0.0160 (9)	−0.0022 (7)	0.0042 (7)	−0.0002 (7)
C14	0.0171 (10)	0.0170 (9)	0.0141 (9)	−0.0004 (7)	0.0029 (7)	−0.0003 (7)
C15	0.0151 (9)	0.0169 (9)	0.0155 (9)	0.0003 (7)	0.0053 (7)	0.0029 (7)
C16	0.0209 (10)	0.0226 (10)	0.0195 (10)	0.0042 (8)	0.0068 (8)	−0.0013 (8)
C17	0.0177 (10)	0.0257 (10)	0.0280 (11)	0.0076 (8)	0.0083 (8)	0.0073 (9)
C18	0.0151 (10)	0.0250 (10)	0.0253 (11)	−0.0001 (8)	0.0009 (8)	0.0060 (8)
C19	0.0262 (12)	0.0360 (12)	0.0306 (12)	0.0019 (10)	0.0156 (10)	0.0064 (9)
C20	0.0335 (12)	0.0182 (9)	0.0225 (10)	0.0016 (9)	0.0107 (9)	0.0003 (8)

### Geometric parameters (Å, º)

**Table:**

S1—C18	1.709 (2)	C8—C9	1.380 (3)
S1—C15	1.7243 (19)	C8—H8A	0.9500
O1—C13	1.248 (2)	C9—C10	1.384 (3)
N1—C7	1.382 (2)	C9—H9A	0.9500
N1—C6	1.417 (2)	C10—C11	1.382 (3)
N1—H1N1	0.85 (3)	C10—H10A	0.9500
N2—C13	1.354 (2)	C11—C12	1.406 (3)
N2—N3	1.382 (2)	C11—H11A	0.9500
N2—H1N2	0.89 (2)	C12—C13	1.475 (3)
N3—C14	1.287 (2)	C14—C15	1.442 (3)
C1—C2	1.381 (3)	C14—H14A	0.9500
C1—C6	1.391 (3)	C15—C16	1.368 (3)
C1—H1A	0.9500	C16—C17	1.415 (3)
C2—C3	1.387 (3)	C16—H16A	0.9500
C2—H2A	0.9500	C17—C18	1.355 (3)
C3—C4	1.385 (3)	C17—H17A	0.9500
C3—H3A	0.9500	C18—H18A	0.9500
C4—C5	1.411 (3)	C19—H19A	0.9800
C4—C19	1.510 (3)	C19—H19B	0.9800
C5—C6	1.399 (3)	C19—H19C	0.9800
C5—C20	1.505 (3)	C20—H20A	0.9800
C7—C8	1.406 (3)	C20—H20B	0.9800
C7—C12	1.417 (3)	C20—H20C	0.9800
			
C18—S1—C15	91.42 (10)	C9—C10—H10A	120.5
C7—N1—C6	125.71 (17)	C10—C11—C12	121.68 (18)
C7—N1—H1N1	115.0 (17)	C10—C11—H11A	119.2
C6—N1—H1N1	117.7 (16)	C12—C11—H11A	119.2
C13—N2—N3	119.15 (15)	C11—C12—C7	119.14 (17)
C13—N2—H1N2	121.9 (15)	C11—C12—C13	119.70 (17)
N3—N2—H1N2	118.9 (15)	C7—C12—C13	121.12 (16)
C14—N3—N2	114.37 (15)	O1—C13—N2	121.08 (17)
C2—C1—C6	120.20 (18)	O1—C13—C12	123.01 (16)
C2—C1—H1A	119.9	N2—C13—C12	115.90 (15)
C6—C1—H1A	119.9	N3—C14—C15	120.51 (17)
C1—C2—C3	119.58 (18)	N3—C14—H14A	119.7
C1—C2—H2A	120.2	C15—C14—H14A	119.7
C3—C2—H2A	120.2	C16—C15—C14	126.67 (17)
C4—C3—C2	120.99 (19)	C16—C15—S1	111.12 (15)
C4—C3—H3A	119.5	C14—C15—S1	121.70 (14)
C2—C3—H3A	119.5	C15—C16—C17	112.75 (18)
C3—C4—C5	120.02 (17)	C15—C16—H16A	123.6
C3—C4—C19	119.07 (18)	C17—C16—H16A	123.6
C5—C4—C19	120.90 (17)	C18—C17—C16	112.26 (18)
C6—C5—C4	118.19 (17)	C18—C17—H17A	123.9
C6—C5—C20	121.03 (17)	C16—C17—H17A	123.9
C4—C5—C20	120.76 (17)	C17—C18—S1	112.46 (16)
C1—C6—C5	120.97 (18)	C17—C18—H18A	123.8
C1—C6—N1	119.99 (17)	S1—C18—H18A	123.8
C5—C6—N1	118.97 (17)	C4—C19—H19A	109.5
N1—C7—C8	121.51 (18)	C4—C19—H19B	109.5
N1—C7—C12	120.46 (17)	H19A—C19—H19B	109.5
C8—C7—C12	117.96 (17)	C4—C19—H19C	109.5
C9—C8—C7	121.33 (19)	H19A—C19—H19C	109.5
C9—C8—H8A	119.3	H19B—C19—H19C	109.5
C7—C8—H8A	119.3	C5—C20—H20A	109.5
C8—C9—C10	120.95 (18)	C5—C20—H20B	109.5
C8—C9—H9A	119.5	H20A—C20—H20B	109.5
C10—C9—H9A	119.5	C5—C20—H20C	109.5
C11—C10—C9	118.90 (18)	H20A—C20—H20C	109.5
C11—C10—H10A	120.5	H20B—C20—H20C	109.5
			
C13—N2—N3—C14	−177.33 (16)	C9—C10—C11—C12	0.1 (3)
C6—C1—C2—C3	−1.3 (3)	C10—C11—C12—C7	1.5 (3)
C1—C2—C3—C4	−0.7 (3)	C10—C11—C12—C13	178.95 (17)
C2—C3—C4—C5	1.6 (3)	N1—C7—C12—C11	−179.49 (18)
C2—C3—C4—C19	−177.56 (18)	C8—C7—C12—C11	−2.7 (3)
C3—C4—C5—C6	−0.4 (3)	N1—C7—C12—C13	3.1 (3)
C19—C4—C5—C6	178.70 (18)	C8—C7—C12—C13	179.91 (17)
C3—C4—C5—C20	−178.59 (18)	N3—N2—C13—O1	13.8 (3)
C19—C4—C5—C20	0.6 (3)	N3—N2—C13—C12	−165.63 (15)
C2—C1—C6—C5	2.5 (3)	C11—C12—C13—O1	−155.40 (18)
C2—C1—C6—N1	179.59 (18)	C7—C12—C13—O1	22.0 (3)
C4—C5—C6—C1	−1.6 (3)	C11—C12—C13—N2	24.0 (2)
C20—C5—C6—C1	176.56 (18)	C7—C12—C13—N2	−158.62 (17)
C4—C5—C6—N1	−178.73 (17)	N2—N3—C14—C15	−170.89 (16)
C20—C5—C6—N1	−0.6 (3)	N3—C14—C15—C16	176.61 (19)
C7—N1—C6—C1	49.4 (3)	N3—C14—C15—S1	5.5 (3)
C7—N1—C6—C5	−133.4 (2)	C18—S1—C15—C16	0.06 (15)
C6—N1—C7—C8	12.2 (3)	C18—S1—C15—C14	172.39 (16)
C6—N1—C7—C12	−171.05 (18)	C14—C15—C16—C17	−171.90 (18)
N1—C7—C8—C9	179.14 (19)	S1—C15—C16—C17	0.0 (2)
C12—C7—C8—C9	2.4 (3)	C15—C16—C17—C18	0.0 (3)
C7—C8—C9—C10	−0.8 (3)	C16—C17—C18—S1	0.1 (2)
C8—C9—C10—C11	−0.5 (3)	C15—S1—C18—C17	−0.07 (16)

### Hydrogen-bond geometry (Å, º)

Cg1 and Cg2 are the centroids of the S1/C15–C18 and C1–C6 rings, respectively.

**Table:**

*D*—H···*A*	*D*—H	H···*A*	*D*···*A*	*D*—H···*A*
N2—H1*N*2···O1^i^	0.89 (2)	1.96 (2)	2.808 (2)	160 (2)
N1—H1*N*1···O1	0.85 (2)	2.02 (3)	2.704 (2)	137 (2)
C1—H1*A*···O1^ii^	0.95	2.58	3.410 (2)	146
C3—H3*A*···*Cg*1^iii^	0.95	2.98	3.732 (2)	137
C9—H9*A*···*Cg*2^iv^	0.95	2.84	3.649 (2)	144

Symmetry codes: (i) *x*, −*y*+1/2, *z*+1/2; (ii) −*x*+1, −*y*, −*z*+2; (iii) −*x*+1, *y*−1/2, −*z*+3/2; (iv) *x*, *y*, *z*+1.
